# Public expenditure on Non-Communicable Diseases & Injuries in India: A budget-based analysis

**DOI:** 10.1371/journal.pone.0222086

**Published:** 2019-09-12

**Authors:** Indrani Gupta, Avantika Ranjan

**Affiliations:** Health Policy Research Unit, Institute of Economic Growth (IEG), New Delhi, India; BITS Pilani, INDIA

## Abstract

**Background:**

Resource allocation decisions for disease categories can be informed by proper estimates of the magnitude and distribution of total spending. In the backdrop of a high burden of Non-Communicable Diseases and Injuries (NCDI) in India, and a paucity of estimates on government spending on NCDI, this paper attempts to analyse public sector expenditure on NCDI spending in India.

**Methods:**

Various recent budget documents of the Centre and States/Union Territories have been used to extract expenditure on NCDI. The aggregates thus arrived at have been analysed to estimate aggregate and state level per capita spending. State level spending have been compared against disease burden using DALYs. Patterns of spending on NCDI across states were also analysed together with state level poverty to observe possible patterns.

**Findings:**

The total spending on NCDI by the government is low at less than 0.5% of GDP. NCDI spending is little more than one-fourth of total health spending of the country and most spending takes place at the state level (80%). The Ministry of Health and Family Welfare’s share in Central spending on NCDI is around 65%, and currently it spends 20% of its total health spending on NCDI. The gap between spending and DALYs is the most for the economically vulnerable states. Also, the states with high poverty levels also have low per capita expenditure on NCDI

**Interpretation:**

India does not depend on donor funding for health. It will have to step up domestic funding to address the increasing disease burden of NCDIs and to reduce the high out-of-pocket expenditure on NCDI. Policies on NCDI need to focus on UHC, service integration and personnel gaps.

## Introduction

In 2016, non-communicable diseases and injuries (NCDIs) comprised 63 percent of all deaths in India, including cardiovascular diseases (27%), chronic respiratory diseases (11%), cancer (9%), diabetes (3%), other NCDs (13%) and injuries (11%) [1]. The prevalence of NCDIs is likely to increase in the coming years due to higher life expectancy as well as factors such as urbanization and industrialization [2].

In face of increasing burden of Non-Communicable Diseases and Injuries (NCDI) in India, it is imperative to put in place a cogent set of interventions to stop a severe impact of the NCDI epidemic on households and the economy. Taking note of the increasing burden of non-communicable diseases, the government initiated a National Programme for Prevention and Control of Cancers, Diabetes, Cardiovascular Diseases and Stroke (NPCDCS) during 2010–11 [3]. The focus of NPCDCS is on promotion of healthy life styles, early diagnosis and management of diabetes, hypertension, cardiovascular diseases & common cancers [4]. While the Plan is timely and impressive, it needs to be backed up by adequate financing. Analysis of state level spending on NCDIs, therefore, is important to understand whether such spending is commensurate with the disease burden of NCDI in the country. An important factor to take into consideration in such analysis is the range of diseases that qualify as non-communicable, besides the most reported ones like cardiovascular diseases, diabetes and cancer.

The overall expenditure on health by the government in India is about 1.1 percent as per the National Health Accounts which naturally sets an upper limit on the potential spending on any one or a group of diseases, including for NCDI [5]. There are no current published estimates available on how much the government spends on NCDI, though there is evidence of increasing out-of-pocket expenditure on non-communicable diseases in India. An earlier attempt to estimate government spending on NCDI found that India spent about 39 percent of total health expenditure on 5 major NCDs in 2004 [6].

This paper attempts to estimate expenditure on NCDI undertaken by the government with budget data pertaining to 2012–13 to 2015–16. It also analyses the expenditure patterns across states in the context of state level spending on NCDI. These estimates can serve as benchmarks for future analysis of NCDI expenditures in the country. Finally, state level spending on NCDI is linked to disability-adjusted-life years (DALY) from NCDI and poverty levels.

In addition to the actual findings, this paper contributes to the health expenditure literature by providing a methodology for extracting data from government budgets for particular diseases. The analysis is based on budgets available in public domain, which lack the granularity required for precise calculations, but are useful for estimating broad aggregates. However, assumptions are required and algorithms needed to estimate the total spending on NCDIs by the government. These approaches can serve as a model for future such estimates, and also for other countries and contexts where disease-specific National Health Accounts are yet to materialize.

A detailed methodology for estimating the total expenditure on NCDI is presented in the next section.

## Materials and methods

NCDI expenditure for the Centre is estimated for 2012–13 to 2016–17; due to data constraints, figures for state and Union Territories (UT) are estimated only for 2015–16. All estimates for the Central government are based on “actual” and are not “budget” or “revised” estimates-terms used in Indian government budgets. The ‘Budget Estimate’ for any ministry or scheme is the amount allocated to it in the budget papers for the following year. In case some ministries require supplementary funds, these are reflected in the revised estimates for the current year. Actual expenditures are the final amounts spent under different heads and may exceed (or fall short of) the Revised Estimates.

### Expenditure by the Central Government

NCDI expenditure for the Centre is calculated from the Union budget documents of various ministries. The key ministry is the Ministry of Health and Family Welfare (MOHFW), for which the Demand for Grants (expenditure budget) has been used [7]. Other ministries with a significant component of health expenditure in them are the Ministries of Defence, Labour and Employment, Mines, Post, Railways and Science and Technology. The total for these other ministries has been taken from MOHFW’s Health Sector Financing by Centre and States/Union Territories (UT’s) [8]^_^.

### Expenditure by the State Governments and UT’s

For each state and UT, health expenditures are taken from the annexure of Centre-State Finance documents available on the MOHFW website for 2015–16 [9].

### Classification of NCDI items

The Global Burden of Disease (GBD) 2015 Cause list has been used to list diseases as Communicable Diseases or Non-Communicable Diseases and Injuries [10]. There are various categories of expenditure in the government’s Demand for Grants. Broadly, expenditure is reported under 6 categories. Those easily identifiable as being for communicable diseases are not included in the calculations. Spending under ‘general’ items which are not disease specific and from which no apportionments have been made to NCDI are also excluded.

Among items that contain NCDI elements one category is those where it is pure NCDI is listed such as cancer, mental health etc. and is taken wholly as NCDI expenditure. The second category is the items related to NHM. For each state the programme Implementation Plans (PIP’s) have been used to apportion NCDI as a percentage of the total NHM in cases where the NHM line item is not specifically mentioned. The third category is of hospitals, medical teaching institutes, research, training etc. from which 70 percent of the expenditures were allocated to NCDI. This was done based on detailed analysis of budgets of India’s premier medical, teaching and research institute—the All India Institute of Medical Sciences (AIIMS); department-wise admissions and discharge data of the institute was used to estimate the percentage of expenditure that can be classified as NCDI expenditure, which came to about 70 percent [11]. In case of hospitals/ teaching institutes related to AYUSH (Ayuveda, Yoga, Unani, Siddha, Homeopathy) half of the expenditure was apportioned to NCDI. Finally, in the absence of any other evidence on how much of spending of other central ministries pertain to NCDIs, we have assigned one-third of the total expenditure to NCDI, though it is likely to be higher, since these comprise reimbursements for medical expenditure of employees.

To arrive at per capita figures, the population of each State, UT, All- India was taken from the Population Projections of the RGI (Registrar general of India) and the average of 2012 and 2013 was taken for arriving at population for 2012–13 [12].

For GDP, values were taken from Reserve Bank of India (RBI) 2011–12 base series [13].

The GSDP (Gross State Domestic Product) for each state was taken from Ministry of Statistics and Planning Implementation’s National Accounts [14].

Purchasing Power Parity (PPP) values were also averaged out to arrive at a figure for 2012–13 [15].

National Health Mission (NHM) NCDI values for the state were arrived at from each of the individual State Programme Implementation Plans (PIPs) given on the NHM website [16]. The ratio of NCD to the total NHM figures were used to arrive at the proportion of NCD spending out of NHM line-items.

### Calculating State level DALYs

We use the Global Health Exchange data of the Institute for Health Metrics and Evaluation (IHME) for 2015 for total DALYs lost for NCDI by adding DALYs lost due to NCD and injuries respectively [17]. Total DALYs thus estimated for each state is then divided by the state population to get per capita DALYs lost for each state.

The percentage of population below poverty line (BPL) has been taken from the Niti Aayog estimates for the year 2011–12, based on Tendulkar methodology [18].

## Results

### All India spending on NCDI

Overall, the total expenditure on NCDI by the Centre and the states/ UTs for 2015–16 was Rs 39843.1 Crores or $229.64 million in PPP. In per capita terms this is Rs 315.86 or $18.2 in PPP. This should be assessed in the context of the rather low per capita health spending of India which was Rs. 1,097 in 2015–16 or $63 in PPP. This indicates that NCDI expenditure is slightly less than 1/3rd of the total health expenditure in the country (29%). As will be observed below, the increase in NCDI spending has happened over the last 3 years, with the government deciding to consciously push NCDI activities in the country via its programme on non-communicable disease under the National Health Mission (NHM).

**Fig 1:**
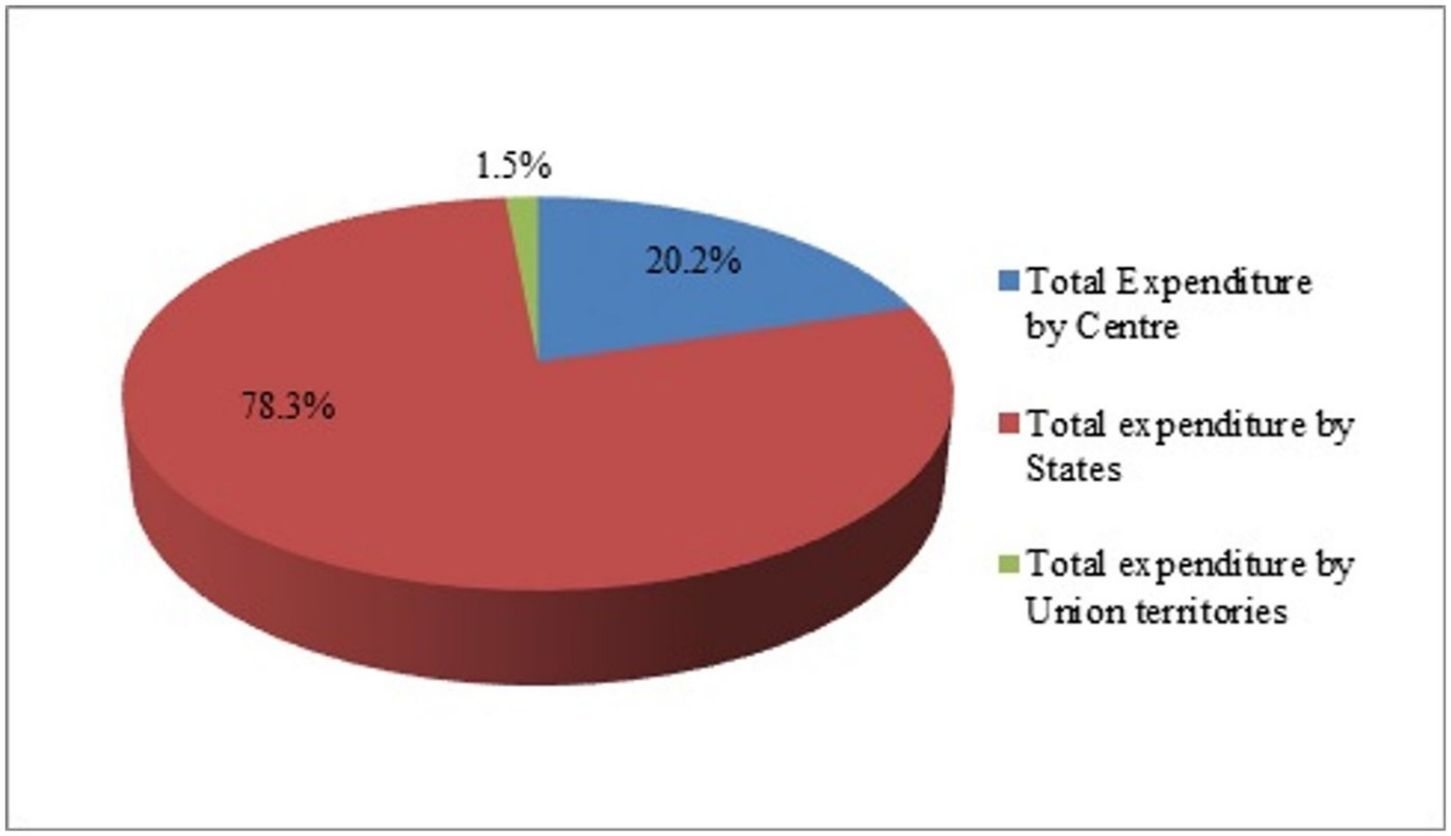
Share of All-India NCDI expenditure-2015-16 actuals.

All India total expenditure on NCDI is $ 229.6 million in PPP. Most of the expenditures on NCDI are taking place at the states and UTs level (about 80%), with the centre spending 20% on NCDIs in the country (Fig 1).

### NCDI spending by the Central Government

**Fig 2:**
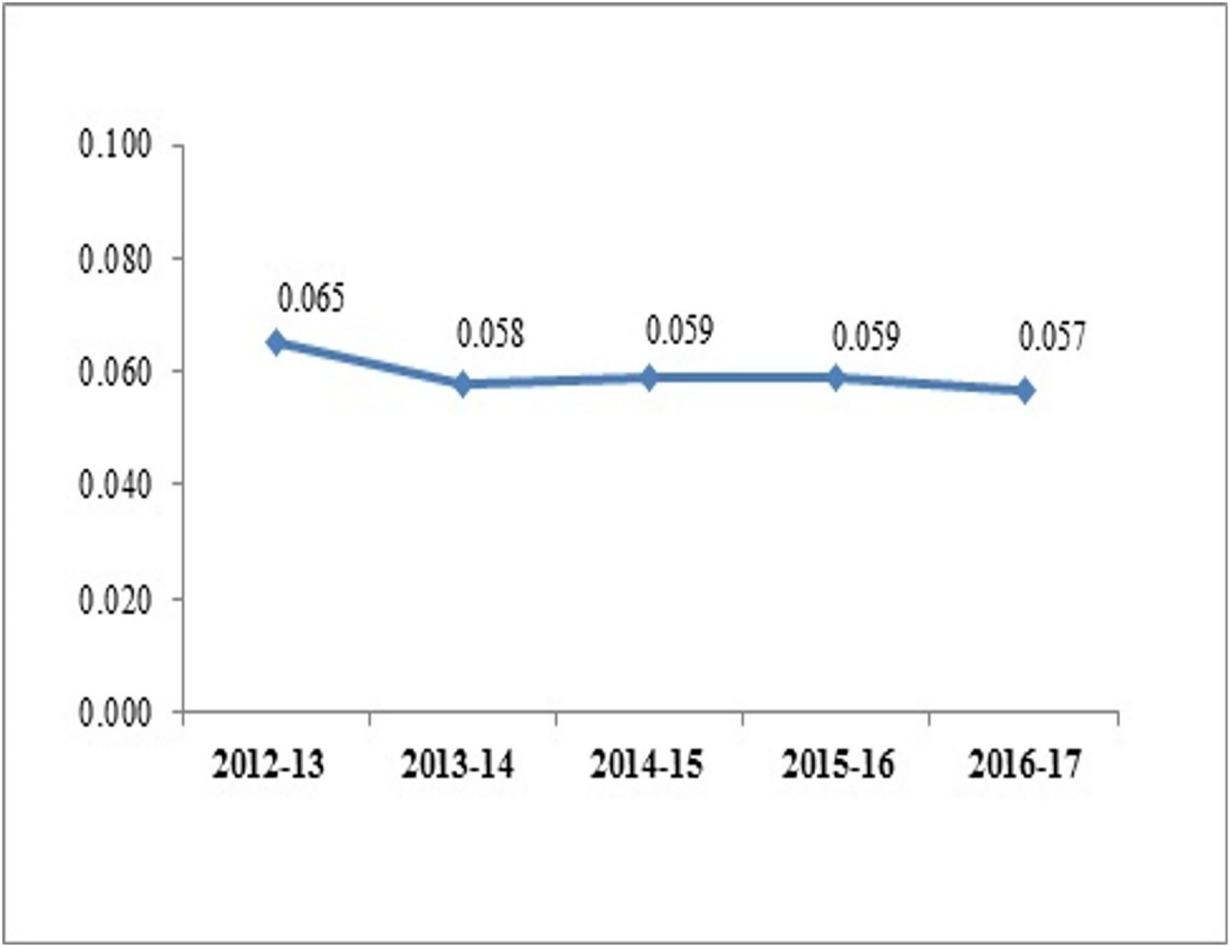
Expenditure on NCDI by Centre as a % of GDP.

For the Centre as a whole, as a percentage of GDP, the share of NCDI is between 0.057–0.65 percent over the years remaining almost constant in the recent years (Fig 2).

**Fig 3:**
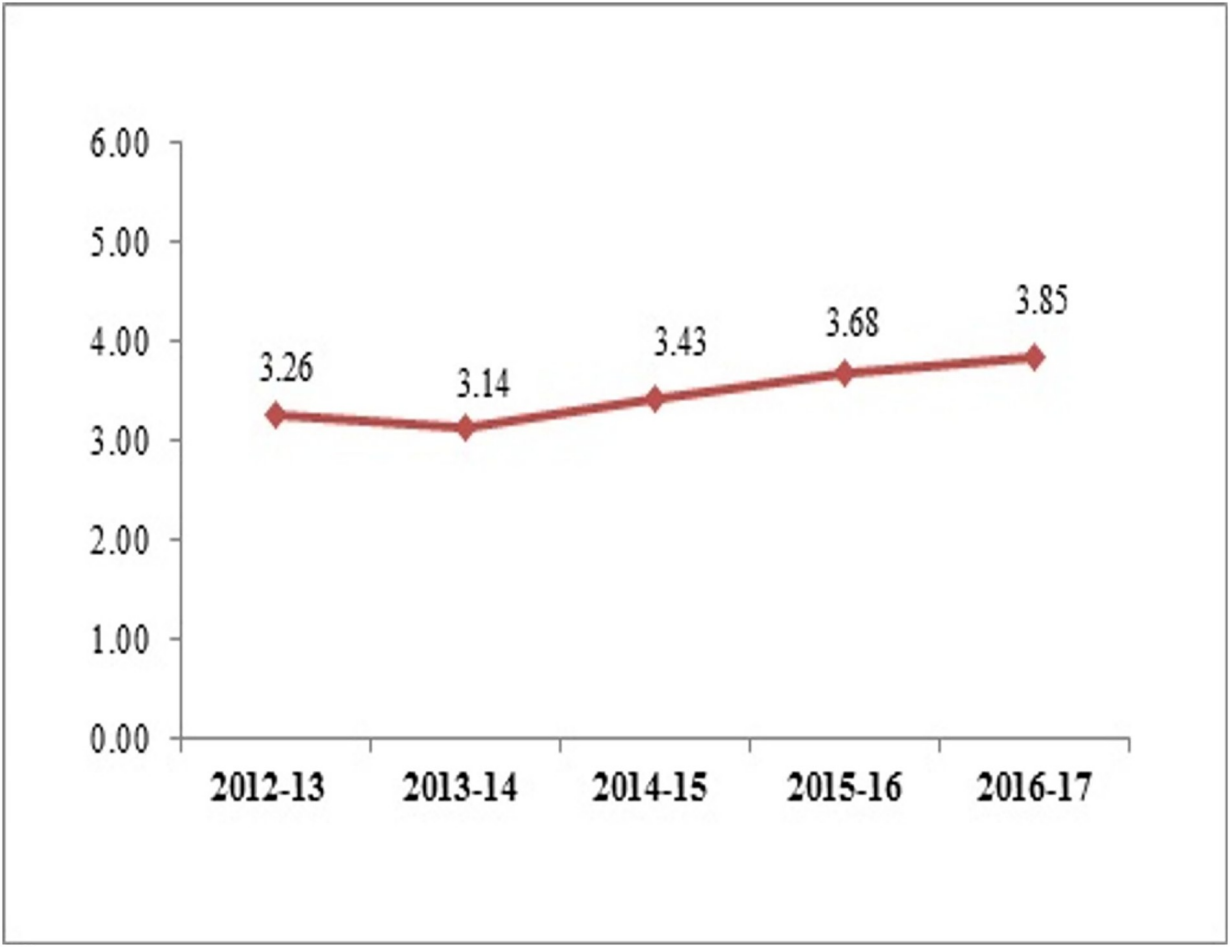
Per capita spending on NCDI by the Centre (PPP $).

In terms of per capita spending in PPP $ on NCDI by the Centre there has been some slight increase from 3.26 to 3.85 in PPP $, within an overall low level of spending (Fig 3).

Among all the Central ministries, the MOHFW spends the majority on NCDI at about 65 percent, with other central ministries spending the remaining 35 percent.

Has the spending on NCDI by MOHFW changed over the last few years?

**Fig 4:**
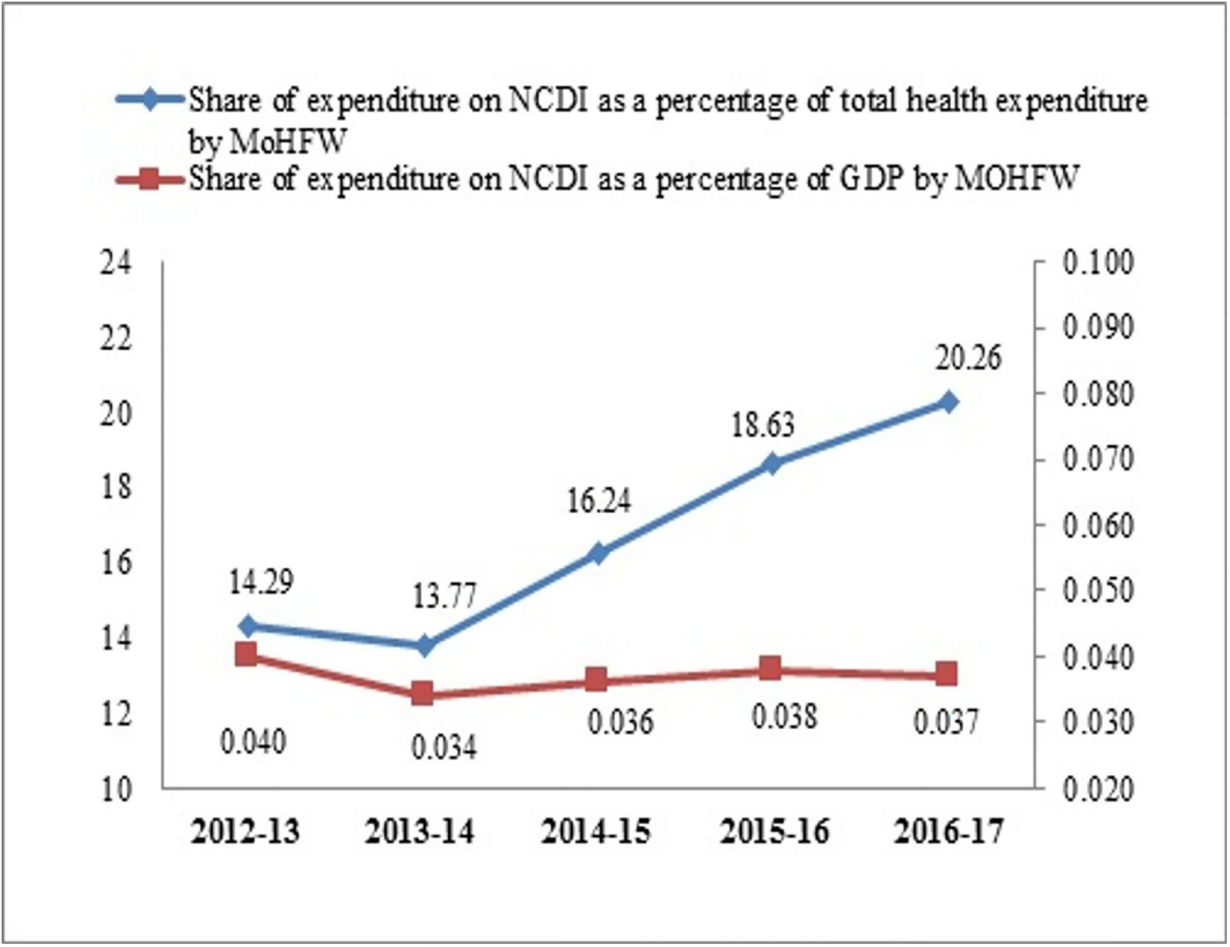
NCDI expenditure by MoHFW.

Recent trends in NCDI expenditure by MOHFW show that the share of NCDI in total health spending has increased steadily over the last 4 years, from 14 to 20 percent between 2012–13 and 2016–17. However, its share in GDP has been negligible (0.034–0.037) despite the slight increase in the last three years (Fig 4).

### NCDI spending by the State Governments

**Fig 5:**
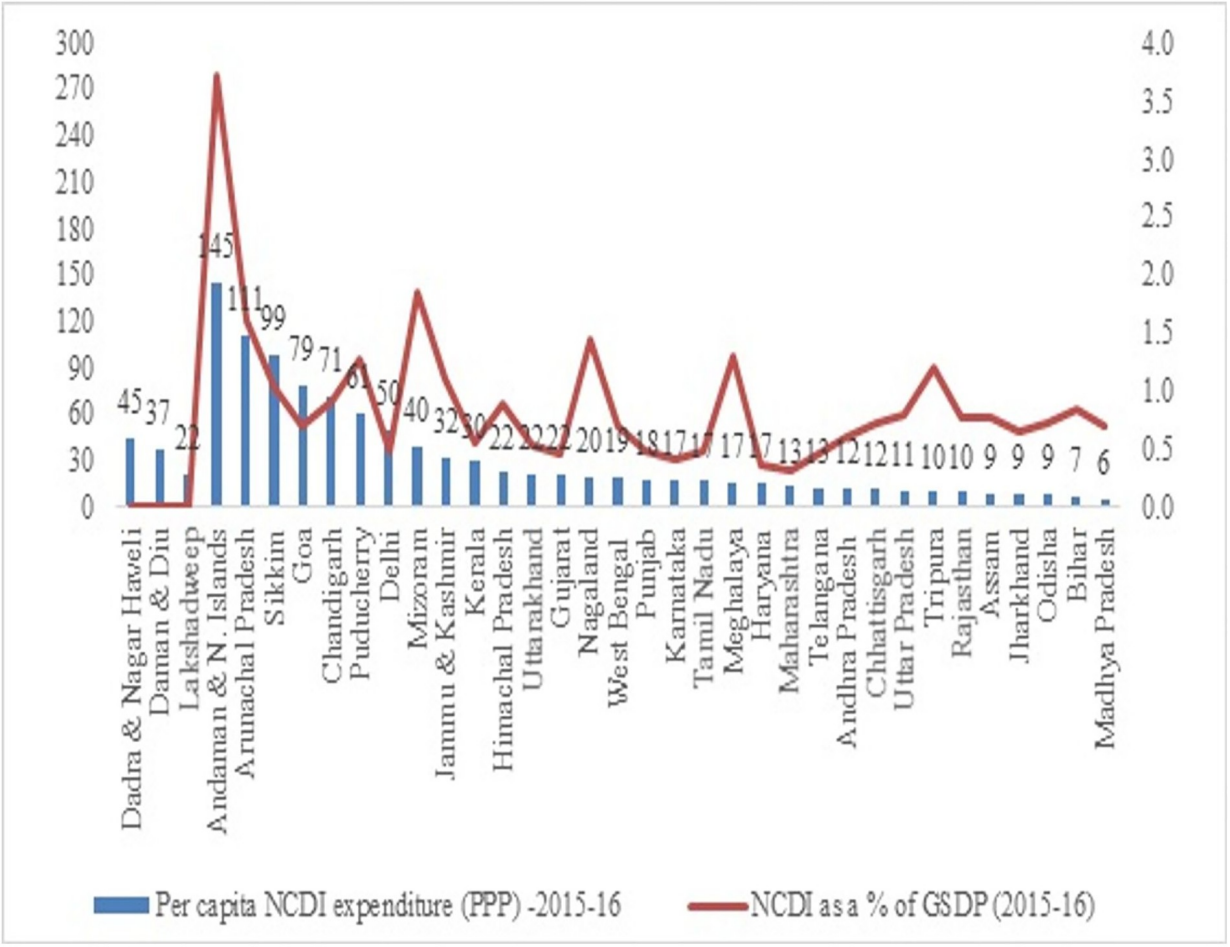
Per capita NCDI expenditure (PPP) and NCDI as a % of GSDP.

The state level variations in NCDI spending is analysed based on per capita expenditure on NCDI and NCDI expenditure as a percentage of GSDP (Gross State Domestic Product). GSDP for Lakshadweep, Dadra & Nagar Haveli, Daman & Diu was unavailable. Also, Manipur was not included in the analysis of state governments because of paucity of data (Fig 5).

There is significant variation on NCDI spending with some of the UTs (ex. Andaman) spending more than 20 times of the lowest spending in the group (ex. Bihar). Generally, the states that have lower per capita expenditure on NCDI are also states that spend less on NCDI as a percentage of their state incomes. Also, the Empowered Action Group states which comprises of eight socio-economically backward states of India and includes the state of Bihar, Chhattisgarh, Jharkhand, Madhya Pradesh, Orissa, Rajasthan, Uttaranchal and Uttar Pradesh plus the state of Assam (together EAG+1) are spending less on NCDI relative to their GSDP. This is more apparent when we look at NCDI expenditure and poverty levels below.

### Poverty, DALYs and expenditure on NCDI

What are the patterns of spending on NCDI across states and does how do these patterns relate to state level poverty?

**Fig 6:**
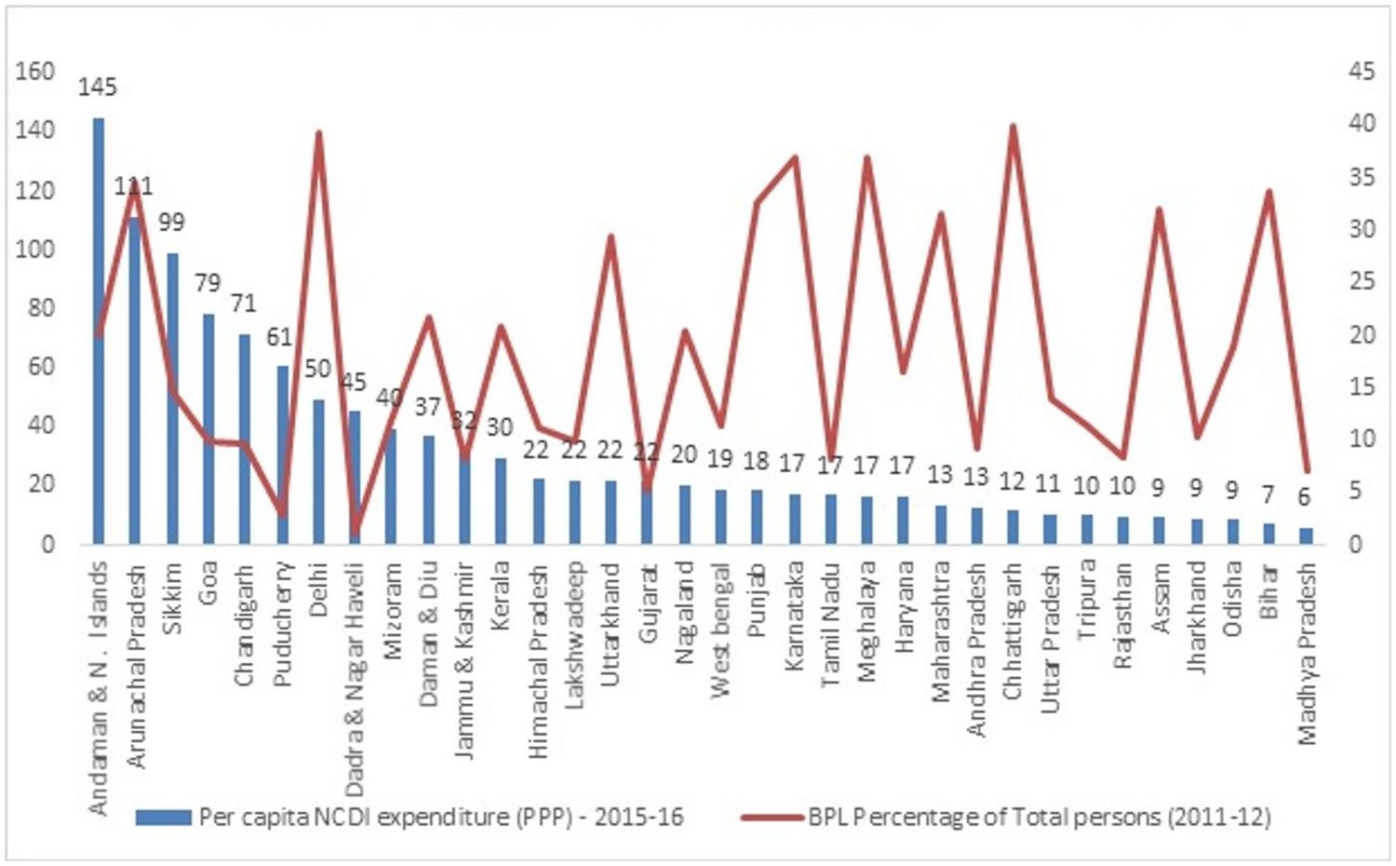
Per capita NCDI expenditure (PPP) v/s BPL percentage of total persons.

In this section per capita expenditure across states is plotted against percentage of below-poverty-line (BPL) population. The general pattern is quite consistent—at least for the major states- with the states with high poverty levels also showing low per capita expenditure on NCDI. Here, Andhra Pradesh is inclusive of Telangana as BPL figures for Telangana are not available. Clearly, poorer states–if also burdened with high prevalence of NCDIs–would be facing major challenges in resource allocations (Fig 6).

**Fig 7:**
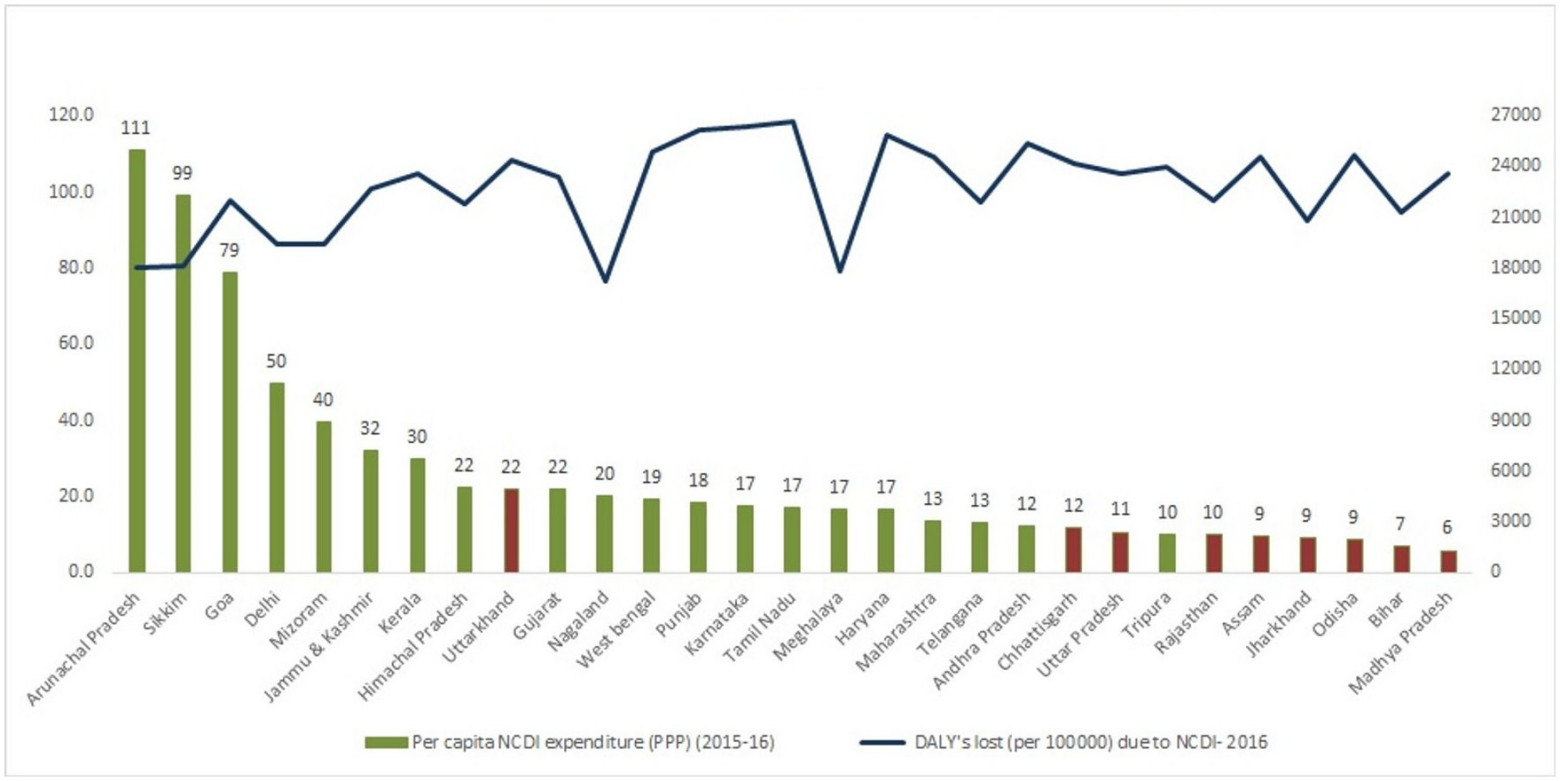
Per capita NCDI expenditure (PPP) and DALY’s lost due to NCDI focusing on EAG+1 states.

Per capita expenditures on NCDI when plotted against per capita DALYs lost for NCDI across states give a picture of gaps in investment in NCDIs across states. DALY’s is DALY’s per 100,000 and DALY’s for individual Union Territories are unavailable (Fig 7).

The gap between spending and DALYs is the most for the EAG+1 states (marked in red), indicating that despite high burden of NCDIs, the states are not being able to spend commensurate amounts on NCDIs, and would need to work the hardest to close the NCDI spending gap at the earliest. The Union Territories are not included in this graph as DALY’s for Union territories are not given individually but as a combined total.

## Discussion

India faces enormous challenges in providing a basic standard of affordable healthcare, and the private sector is the major player in both financing and delivery of healthcare. Twenty-six percent of the total healthcare expenditure was general government funding, which is much lower than the average of forty-six percent for the South East Asia region [19]. India is among a group of countries that have the lowest levels of public investment in healthcare. Households face high financial burden in India, with 62.5 percent of total health expenditure comprising out-of-pocket payments in 2014–15 according to the National Health Accounts estimates [5]. This is likely to impose a severe burden on households due to the high cost of treatment of NCDIs; the current expenditure by households (including prepayments for insurance premiums) is estimated to be 66.3 percent of total health expenditure [5]. It will also pose resource allocation and prioritization challenges to the policymakers not only within the group of diverse diseases that comprise NCDI, but also across the whole gamut of diseases, including communicable and re-emerging diseases.

The government has increased its NCDI spending under the NHM–which runs as a centre-state resource share model—under the head ‘Flexible Pool for Non-Communicable Diseases’. This has ensured greater funding on NCDIs by the states to match the central transfers under the NHM. However, as the analysis indicates, these resources will not be sufficient and the states will need to put in much greater efforts to tackle NCDIs. However, any reallocation with a constant resource envelope will likely squeeze out investment in other essential areas like communicable diseases, re-emerging and newer diseases as well as for health systems strengthening. State allocations for health have not been improving significantly over the years, especially for the EAG states. States that have high burden of NCDI as well as significant poverty would require extra push to enable them to step up financing for NCDIs. It is not immediately apparent how the states would increase their health spending. Also, there are no estimates available to indicate the NCDI spending gaps in the states. The financing gap for NCDIs is not only limited to India; data indicates that non-communicable diseases account for 67 percent of deaths in low- and middle-income countries but receive only 1 percent of health funding [20].

India is self-sufficient in the health sector and does not depend on donor funding for most of its programmes. Clearly, funding will have to be domestically raised for NCDI within a larger resource envelope for health in general. Areas that will need particular focus would be management and human resources. Studies have found that India is ill-prepared at the primary care level to tackle with diseases like diabetes and hypertension [21]. In the absence of volume and quality of NCDI care, out-of-pocket expenditures would continue to rise rapidly due to the high costs of treatment of NCDI and their chronic nature [22]. Existing health coverage programmes are not comprehensive enough to reduce this burden, and the huge infrastructural and personnel gaps in rural India in government facilities continue to result in most NCDI treatments being done at private providers and facilities [23].

Given the large number of conditions that go into NCDI, there is an urgent need to think of a more comprehensive approach to tackle NCDIs. While India’s spending on NCDI seems comparable or even higher than other countries (for example, Mongolia spends 34% of total health expenditure on 4 conditions, but 2/3rds of this is out-of-pocket), the very modest overall resource envelope for health makes the level of spending very low [24].

The lack of data is one major concern and prevents comparisons across countries. There have been efforts to decompose National Health Accounts into disease categories, but these efforts have been very few [25]. The disease-specific NHA can be given more attention in India as well, so that baseline and comparable data on spending on NCDIs can be generated.

At the same time, continuous pressure on the government to increase funding for health for proven interventions should be kept up to avoid inefficient use of scarce resources and to ensure that funding does not decline on communicable diseases and other public health priorities. It is imperative to explore innovative funding by tapping private corporate world and other sources like ear-marked funding. The government might consider setting up a high level body that can address the diverse planning challenges that are posed by the group of diseases under NCDI, draw up a strategic plan on financing, personnel and service delivery, and in general prepare a road map–including for financing and its possible sources—for tackling NCDIs in India.

## Supporting information

S1 Table All-India NCDI expenditure-2015-16 actuals. (DOCX)

S2 Table Expenditure on NCDI by the Central Government as a % of GDP. (DOCX)

S3 Table Per capita spending on NCDI by the Central Government in PPP $. (DOCX)

S4 Table Details of NCDI expenditure by MoHFW. (DOCX)

S5 Table State-wise details of Per capita NCDI expenditure (PPP) and NCDI as a % of GSDP. (DOCX)

S6 Table State-wise details of Per capita NCDI expenditure (PPP) v/s BPL percentage of total persons (2011–12). (DOCX)

S7 Table State-wise details of Per capita NCDI expenditure (PPP) and DALY’s lost (per 100000) due to NCDI with a focus on EAG+1 states. (DOCX)
