# Supplementary material for: Public expenditure on Non-Communicable Diseases & Injuries in India: A budget-based analysis
Source: PLoS One. 2019 Sep 12;14(9):e0222086. doi: 10.1371/journal.pone.0222086 (PMC6742225; doi:10.1371/journal.pone.0222086)
Supplement: S1 Table — (DOCX) [file pone.0222086.s001.docx]

| **NCDI Expenditure** | **2015-16 Actuals (in Crores of rupees)** |
| --- | --- |
| Total Expenditure by Centre | 8045.76 |
| Total expenditure by States | 31213.21 |
| Total expenditure by Union territories | 584.10 |
