# Supplementary material for: Public expenditure on Non-Communicable Diseases & Injuries in India: A budget-based analysis
Source: PLoS One. 2019 Sep 12;14(9):e0222086. doi: 10.1371/journal.pone.0222086 (PMC6742225; doi:10.1371/journal.pone.0222086)
Supplement: S2 Table — (DOCX) [file pone.0222086.s002.docx]

|  | **Detailed Entries For Expenditure on NCDI (In Rs Crores)** | **2012-13** | **2013-14** | **2014-15** | **2015-16** | **2016-17** |
| --- | --- | --- | --- | --- | --- | --- |
| 1 | Ministry of Health & Family welfare | 3984.28 | 3838.44 | 4527.63 | 5193.84 | 5649.03 |
| 2 | Other Central Ministries | 2499.54 | 2666.92 | 2775.18 | 2851.92 | 2989.74 |
|  | **TOTAL Expenditure by Centre on NCDI (in Crores)** | **6483.82** | **6505.36** | **7302.81** | **8045.76** | **8638.77** |
|  | **GDP values in Rs Crores** | 9944013 | 11233522 | 12445128 | 13682035 | 15183709 |
|  | **Expenditure on NCDI by Centre as a % of GDP** | **0.065** | **0.058** | **0.059** | **0.059** | **0.057** |
