# Supplementary material for: Public expenditure on Non-Communicable Diseases & Injuries in India: A budget-based analysis
Source: PLoS One. 2019 Sep 12;14(9):e0222086. doi: 10.1371/journal.pone.0222086 (PMC6742225; doi:10.1371/journal.pone.0222086)
Supplement: S3 Table — (DOCX) [file pone.0222086.s003.docx]

|  | **Detailed Entries For Expenditure on NCDI (In Rs Crores)** | **2012-13** | **2013-14** | **2014-15** | **2015-16** | **2016-17** |
| --- | --- | --- | --- | --- | --- | --- |
| 1 | Ministry of Health & Family welfare | 3984.28 | 3838.44 | 4527.63 | 5193.84 | 5649.03 |
| 2 | Other Central Ministries | 2499.54 | 2666.92 | 2775.18 | 2851.92 | 2989.74 |
|  | **TOTAL Expenditure by Centre on NCDI (in Crores)** | **6483.82** | **6505.36** | **7302.81** | **8045.76** | **8638.77** |
|  | **Population India in Crores** | 121.58 | 123.12 | 124.65 | 126.14 | 127.62 |
|  | **Per capita spending on NCDI by the Centre in Rs.** | 53.33 | 52.84 | 58.59 | 63.78 | 67.69 |
|  | **PPP conversion factor $** | 16.35 | 16.85 | 17.10 | 17.35 | 17.60 |
|  | **Per capita spending on NCDI by the Centre in PPP $** | 3.26 | 3.14 | 3.43 | 3.68 | 3.85 |
