# Supplementary material for: Public expenditure on Non-Communicable Diseases & Injuries in India: A budget-based analysis
Source: PLoS One. 2019 Sep 12;14(9):e0222086. doi: 10.1371/journal.pone.0222086 (PMC6742225; doi:10.1371/journal.pone.0222086)
Supplement: S4 Table — (DOCX) [file pone.0222086.s004.docx]

| **YEAR** | **Share of expenditure on NCDI as a percentage of total health expenditure by MoHFW** | **Share of expenditure on NCDI as a percentage of GDP by MOHFW** |
| --- | --- | --- |
| **2012-13** | **14.29** | **0.040** |
| **2013-14** | **13.77** | **0.034** |
| **2014-15** | **16.24** | **0.036** |
| **2015-16** | **18.63** | **0.038** |
| **2016-17** | **20.26** | **0.037** |
