# Supplementary material for: Public expenditure on Non-Communicable Diseases & Injuries in India: A budget-based analysis
Source: PLoS One. 2019 Sep 12;14(9):e0222086. doi: 10.1371/journal.pone.0222086 (PMC6742225; doi:10.1371/journal.pone.0222086)
Supplement: S5 Table — (DOCX) [file pone.0222086.s005.docx]

| **STATECODE** | **Per capita NCDI expenditure (PPP) -2015-16** | **NCDI as a % of GSDP (2015-16)** |
| --- | --- | --- |
| Dadra & Nagar Haveli | 45 | 0.00 |
| Daman & Diu | 37 | 0.00 |
| Lakshadweep | 22 | 0.00 |
| Andaman & N. Islands | 145 | 3.71 |
| Arunachal Pradesh | 111 | 1.62 |
| Sikkim | 99 | 1.03 |
| Goa | 79 | 0.71 |
| Chandigarh | 71 | 0.92 |
| Puducherry | 61 | 1.27 |
| Delhi | 50 | 0.48 |
| Mizoram | 40 | 1.84 |
| Jammu & Kashmir | 32 | 1.11 |
| Kerala | 30 | 0.55 |
| Himachal Pradesh | 22 | 0.89 |
| Uttarakhand | 22 | 0.53 |
| Gujarat | 22 | 0.46 |
| Nagaland | 20 | 1.45 |
| West Bengal | 19 | 0.71 |
| Punjab | 18 | 0.49 |
| Karnataka | 17 | 0.42 |
| Tamil Nadu | 17 | 0.48 |
| Meghalaya | 17 | 1.31 |
| Haryana | 17 | 0.36 |
| Maharashtra | 13 | 0.31 |
| Telangana | 13 | 0.47 |
| Andhra Pradesh | 12 | 0.60 |
| Chhattisgarh | 12 | 0.72 |
| Uttar Pradesh | 11 | 0.81 |
| Tripura | 10 | 1.21 |
| Rajasthan | 10 | 0.76 |
| Assam | 9 | 0.77 |
| Jharkhand | 9 | 0.65 |
| Odisha | 9 | 0.73 |
| Bihar | 7 | 0.85 |
| Madhya Pradesh | 6 | 0.70 |
