# Supplementary material for: Public expenditure on Non-Communicable Diseases & Injuries in India: A budget-based analysis
Source: PLoS One. 2019 Sep 12;14(9):e0222086. doi: 10.1371/journal.pone.0222086 (PMC6742225; doi:10.1371/journal.pone.0222086)
Supplement: S6 Table — (DOCX) [file pone.0222086.s006.docx]

| **STATECODE** | **Per capita NCDI expenditure (PPP) - 2015-16** | **BPL Percentage of Total persons (2011-12)** |
| --- | --- | --- |
| Andaman & N. Islands | 145 | 20 |
| Arunachal Pradesh | 111 | 34.7 |
| Sikkim | 99 | 14.7 |
| Goa | 79 | 9.9 |
| Chandigarh | 71 | 9.7 |
| Puducherry | 61 | 2.8 |
| Delhi | 50 | 39.3 |
| Dadra & Nagar Haveli | 45 | 1 |
| Mizoram | 40 | 11.9 |
| Daman & Diu | 37 | 21.8 |
| Jammu & Kashmir | 32 | 8.1 |
| Kerala | 30 | 20.9 |
| Himachal Pradesh | 22 | 11.2 |
| Lakshwadeep | 22 | 9.9 |
| Uttarkhand | 22 | 29.4 |
| Gujarat | 22 | 5.1 |
| Nagaland | 20 | 20.4 |
| West bengal | 19 | 11.3 |
| Punjab | 18 | 32.6 |
| Karnataka | 17 | 37 |
| Tamil Nadu | 17 | 8.2 |
| Meghalaya | 17 | 36.9 |
| Haryana | 17 | 16.6 |
| Maharashtra | 13 | 31.6 |
| Andhra Pradesh | 13 | 9.2 |
| Chhattisgarh | 12 | 39.9 |
| Uttar Pradesh | 11 | 14 |
| Tripura | 10 | 11.3 |
| Rajasthan | 10 | 8.3 |
| Assam | 9 | 32 |
| Jharkhand | 9 | 10.3 |
| Odisha | 9 | 18.9 |
| Bihar | 7 | 33.7 |
| Madhya Pradesh | 6 | 7.1 |
