# Supplementary material for: Public expenditure on Non-Communicable Diseases & Injuries in India: A budget-based analysis
Source: PLoS One. 2019 Sep 12;14(9):e0222086. doi: 10.1371/journal.pone.0222086 (PMC6742225; doi:10.1371/journal.pone.0222086)
Supplement: S7 Table — (DOCX) [file pone.0222086.s007.docx]

| **STATECODE** | **Per capita NCDI expenditure (PPP) (2015-16)** | **DALY's lost (per 100000) due to NCDI- 2016** |
| --- | --- | --- |
| Andaman & N. Islands | 145.0 | 0.00 |
| Chandigarh | 71.4 | 0.00 |
| Puducherry | 60.5 | 0.00 |
| Dadra & Nagar Haveli | 45.3 | 0.00 |
| Daman & Diu | 37.0 | 0.00 |
| Lakshwadeep | 22.2 | 0.00 |
| Arunachal Pradesh | 111.1 | 18041.90 |
| Sikkim | 99.0 | 18169.90 |
| Goa | 78.7 | 21983.16 |
| Delhi | 49.6 | 19428.75 |
| Mizoram | 39.6 | 19380.11 |
| Jammu & Kashmir | 32.0 | 22691.47 |
| Kerala | 29.7 | 23578.16 |
| Himachal Pradesh | 22.5 | 21785.16 |
| Uttarkhand | 22.0 | 24344.65 |
| Gujarat | 21.7 | 23447.54 |
| Nagaland | 20.2 | 17226.51 |
| West bengal | 19.1 | 24892.27 |
| Punjab | 18.4 | 26217.94 |
| Karnataka | 17.4 | 26421.03 |
| Tamil Nadu | 17.0 | 26691.95 |
| Meghalaya | 16.7 | 17884.04 |
| Haryana | 16.5 | 25867.13 |
| Maharashtra | 13.5 | 24628.82 |
| Telangana | 12.9 | 21884.13 |
| Andhra Pradesh | 12.3 | 25353.86 |
| Chhattisgarh | 11.7 | 24184.07 |
| Uttar Pradesh | 10.7 | 23552.12 |
| Tripura | 10.0 | 23959.43 |
| Rajasthan | 9.8 | 21985.68 |
| Assam | 9.4 | 24570.04 |
| Jharkhand | 8.9 | 20768.76 |
| Odisha | 8.7 | 24683.13 |
| Bihar | 7.0 | 21270.76 |
| Madhya Pradesh | 5.8 | 23571.24 |

#Figures on the IHME website are updated regularly. These were the numbers last accesses by the author.
